# KDF1 Promoted Proliferation and Metastasis of Epithelial Ovarian Cancer *via* Wnt/Beta-Catenin Pathway: TCGA-Based Data Mining and Experimental Validation

**DOI:** 10.3389/fgene.2022.808100

**Published:** 2022-02-25

**Authors:** Changyu Zhu, Yilong Liu, Rongsheng Tong, Jianmei Guan

**Affiliations:** ^1^ Department of Pharmacy, Sichuan Academy of Medical Science and Sichuan Provincial People’s Hospital, School of Medicine, University of Electronic Science and Technology of China, Chengdu, China; ^2^ Personalized Drug Therapy Key Laboratory of Sichuan Province, University of Electronic Science and Technology of China, Chengdu, China; ^3^ Central Sterile Supply Department, Sichuan Academy of Medical Sciences and Sichuan Provincial People’s Hospital, Chengdu, China

**Keywords:** TCGA, ovarian cancer, proliferation, KDF1, Wnt

## Abstract

**Objectives:** It has been reported that keratinocyte differentiation factor 1 (KDF1) was related to proliferation, differentiation, and cell cycle. However, the role of KDF1 has not been reported in ovarian cancer. The present study investigated the function and the potential mechanism of KDF1 in ovarian cancer.

**Methods:** We evaluated the prognostic value in ovarian cancer based on data from the Cancer Genome Atlas (TCGA) database. The Kruskal–Wallis test, Wilcoxon signed-rank test, and logistic regression were used to evaluate the relationship between KDF1 expression and clinicopathologic features. The Cox regression and the Kaplan–Meier method were adopted to evaluate prognosis-related factors. Gene Ontology (GO), Kyoto Encyclopedia of Genes and Genomes (KEGG) gene enrichment analysis, and Gene Set Enrichment Analysis (GSEA) were performed to identify the key biological process related to KDF1. Then the expression of KDF1 in ovarian cancer tissues was validated by streptavidin–peroxidase (SP) immunohistochemistry. The proliferation and invasion ability of KDF1 were determined by EdU and Transwell assay, respectively, with KDF1 gene silencing and overexpression. The mRNA expression of KDF1 was determined by qPCR. The protein expression of KDF1 was determined using the Western blot.

**Methods:** By performing differential expression analysis on the ovarian cancer data of the TCGA database, it was found that KDF1 is highly expressed in ovarian cancer patients and associated with poorer overall survival (OS) and progression-free survival (PFS) of ovarian cancer patients. The highly expressed KDF1 may reduce cell adhesion according to GO, KEGG, and GSEA results. After analysis combining the relevant clinical features, we found that the high expression of KDF1 is an independent prognostic factor of ovarian cancer and associated with platinum resistance and tumor metastasis in ovarian cancer. At the same time, the BioGRID database showed that there might be protein–protein interaction between KDF1 and E-cadherin. Then we further validated that the high expression of KDF1 had a close correlation with the stage and grade of ovarian cancer in ovarian cancer tissue chips. Silencing KDF1 inhibited the proliferation and invasion ability of SKOV3 cells. By contrast, ectopic expression of KDF1 promoted the proliferation and invasion ability of A2780 cells. We also found that KDF1 can interact with E-cadherin and regulate the expression of Wnt5A and β-catenin, hence activating Wnt/β-catenin pathway via *in vitro* and vivo experiments.

**Conclusions: **Based on the bioinformatics analysis, *in vitro* experiments, and an *in vivo* study, it is indicated that KDF1 played an important role in ovarian cancer progression and might be a therapeutic target for patients with ovarian cancer.

## Introduction

Ovarian cancer (OV) is the only gynecological tumor among the five leading causes of death in women. According to the statistics report, there were 21,750 new cases and 13,940 deaths in the United States in 2020 (Siegel et al., 2020). According to the tumor-initiating cell type, ovarian cancer was divided into three categories: epithelial cancer, interstitial cancer, and germ cell cancer (Nguyen et al., 2019). Epithelial ovarian cancer (EOC) was the most common ovarian malignancy, accounting for more than 90% of all ovarian cancer. Most patients with ovarian cancer are asymptomatic, and the volume of the ovary is relatively small, the anatomical position of which is deep in the pelvic cavity. Patients often cannot observe specific symptoms early, resulting in the difficulty of early screening of the disease. Therefore, more than 70% newly diagnosed patients were at the advanced stage, resulting in a 5-year survival rate of less than 20%.

Based on the epidemiological characteristics of ovarian cancer, early diagnosis and treatment are very important. However, compared to the increasingly advanced detection technology of other tumors, there has been no reliable screening biomarker and therapeutic target for ovarian cancer so far (Menon et al., 2018).

Keratinocyte differentiation factor 1 (*KDF1*), a protein-coding gene containing a domain of unknown function (DUF4656), was first identified as an essential regulator of the proliferation differentiation decision in epidermal progenitor cells. *Kdf1* was found in mice for the first time by the positive genetic method with the function of retarding proliferation through its inhibition of p63.

Shamseldin et al. (2017)had reported that *KDF1* is silenced in a multigenerational family with ectodermal dysplasia. When the *Kdf1* gene was knocked out, the mouse represented the recapitulation of the phenotype (Shamseldin et al., 2017). Recently, a study reported that *KDF1* was a new candidate gene for non-syndromic tooth agenesis (Zeng et al., 2019). Also, *Kdf1* induced epidermal progenitor cell differentiation through interaction with the cell cycle regulator stratifin (Lee et al., 2013). Many recent studies have shown that stratifin is a critical marker in the process of tumor progression (Shiba-Ishii et al., 2019). The genetic interaction between Kdf1 and more widely studied stratifin (Shamseldin et al., 2017) proves that KDF1 may be related to tumor progression.

At the same time, quantitative proteomics was used to study the interaction group of KDF1. Mass spectrometry had identified that KDF1 could specifically bind to IκB kinase α (IKKα) in differentiated keratinocytes and mediate the regulation of deubiquitination on epidermal differentiation (Li et al., 2020). As a mature component of the NF-κB signaling pathway, IKKα also plays a vital role in cancer progression (Liu et al., 2008; Yang et al., 2016).

Although studies have shown that KDF1 is associated with tumor-related molecules, so far, the expression and function of KDF1 in epithelial ovarian cancer have not been reported. Therefore, in this research, we combined bioinformatics and experimental study to investigate the expression pattern and function of KDF1 in epithelial ovarian cancer and explore the value of KDF1 in EOC diagnosis and treatment.

## Materials and Methods

### Data Acquisition and Preprocessing

The HTSeq-FPKM dataset was downloaded from the GDC TCGA Ovarian Cancer queue of UCSC XENA (https://xenabrowser.net/datapages/), which includes 379 ovarian cancer RNA seq-data. Then the clinical data were downloaded from the TCGA database (Blum et al., 2018) (https://portal.gdc.cancer.gov/) until 12 July 2020. Then the TRAIL TOIL RSEM fpkm (*n* = 7,862) UCSC Toil RNA-seq Recompute dataset was downloaded from the GTEx queue of UCSC XENA. The sequencing data of 88 normal ovarian tissues and 379 ovarian cancer tissues were extracted and analyzed. The different KDF1 expression between OC and non-tumor tissue was also investigated in three RNAseq datasets (GSE12470, GSE18520, and GSE66957), which were downloaded from the GEO database (http://www.ncbi.nlm.nih.gov/geo).

### Differentially Expressed Gene Analysis

We used unpaired Student’s *t*-test within the DESeq2 R package (4.0.0) (Love et al., 2014) to compare the expression data (HTseq-Counts) between high and low expression groups according to the median *kdf1* expression level. The thresholds for the DEGs were |log2-fold change (FC)| >2.0 and adjusted *p* < 0.05.

### Enrichment Analysis and Protein–Protein Interaction Analysis

ClusterProfiler package in R (4.0.0) (Yu et al., 2012) was used to perform Gene Ontology (GO) analysis and Kyoto Encyclopedia of Genes and Genomes (KEGG) analysis to detect the possible function of KDF1. Samples were divided into high and low expression groups according to the expression level of KDF1 expression. As a computational method, the GSEA determines whether *a priori* defined set of genes has statistical significance and concordant differences in two biological states. We performed GSEA between high- and low-KDF1 groups by GSEA Desktop Application (v4.0.3; Broad Institute, Inc., Cambridge, MA, United States). Additionally, the adjusted P and normalized enrichment score (NES) were utilized to sort the enriched pathways in each phenotype (Subramanian et al., 2005). c2. cp.v7.0. symbols.gmt (Curated) in MSigDB Collections was selected as a reference gene set. Gene sets with a false discovery rate (FDR) < 0.25 and adjusted *p* < 0.05 were considered significantly enriched. The KDF1 interacting protein was predicted by Biogrid and visualized by Cytoscape.

### Immunochemistry

This study was approved by the Ethics Committee of Sichuan Provincial People’s Hospital. The approval number issued by the Ethics Committee was as follows: Ethic review (fundamental research) No. 109 of 2016. The location and expression of KDF1 in ovarian cancer and normal ovarian tissues were detected by immunohistochemistry. The ovarian cancer tissue chip was purchased from Alenabio with a total of 110 cases. The ovarian cancer tissue chip contains 80 cases of EOC tissue, and the normal ovarian tissue chip includes 30 samples of normal ovarian tissue. The immunohistochemistry kit and DAB kit were purchased from Zsbio. The procedure of the immunohistochemistry experiment refers to that of previous research. The immunohistochemical score (ranging from 0 to 9) was calculated by multiplying the intensity and percentage scores. Staining intensity was graded on a 0–3 scale: 0, absence of staining; 1, weakly stained; 2, moderately stained; and 3, intensely stained. The percentage of positive tumor cells was scored as follows: 0, absence of tumor cells; 1, <33% of tumor cells; 2, 33–66% of tumor cells; and 3, >66% of tumor cells (Mustapar et al., 2020).

### Cell Culture, Transfection Procedure, and Reagents

A2780 and SKOV3 cells were cultured in DMEM (Sigma, D5796). The medium included streptomycin and 10% fetal bovine serum. The cells were incubated under 5% CO_2_ and 37°C. KDF1 interference vector (called LV3-NC, LV3-shKDF1-1, and LV3-shKDF1-2) and overexpression vector (called LV5-NC and LV5-KDF1) carried by the lentivirus were from Genepharma. The following was the siRNA sequence targeting KDF1. LV3-shKDF1-1: 5′-GUU​UGU​AAG​UAC​AAA​GGU​AA-3'; LV3-shKDF1-2: 5′-GCU​GAU​GUU​CUG​UAU​CUU​AAC-3′ and NC (negative control) siRNA: 5′-UUC​UUC​GAA​GGU​GUC​ACG​UTT-3′.

### Scratch Assay

The scratch assay was performed as in a previous study (Liang et al., 2007). Cells were cultured in a six-well plate to a confluent monolayer. We used a 10 μL pipette tip to scrape the cell monolayer in a straight line vertically. The debris was then removed by washing the cells thrice with PBS (Boster, Wuhan) and replaced with 2 ml of the original medium. Then the images of cells were captured 48 h after scratch.

### EdU Assay

The cell proliferation ability was measured using the EdU experiment. The EdU kit was purchased from GeneCopoeia. The experimental procedures refer to those of the previous literature (Fu et al., 2020).

### RT-qPCR

The qPCR steps mainly include RNA extraction and reverse transcription into cDNA and qPCR. The experimental procedures can be referred to those in the previous literature (Han et al., 2017). The primers were synthesized by Genepharma.

### Western Blot

The expression of KDF1, GAPDH, E-cadherin, and β-catenin was detected by the Western blot. The primary antibody used in the present research included anti-KDF1 (Abcam, ab224760), anti-GAPDH (Abcam, ab181602), anti-E-cadherin (Abcam, ab40772), anti-Wnt5A (Abcam, ab179824), and anti-β-catenin (Abcam, ab223075). Primary antibodies were diluted in Dilution Buffer (Beyotime, P0256) and incubated overnight at 4°C. We used the gel imaging system to analyze the band density and compare it with the internal control.

### Matrigel Invasion Assay

The Matrigel invasion assay was performed to assess cellular invasion ability according to the previous study (Han et al., 2017). 1 × 10^5^ cells were seeded into the upper chamber. After 24 h, the cells on the lower surface of the membrane were fixed with 4% paraformaldehyde and stained with 0.5% crystal solution. Then, the cells were counted and photographed using a microscope.

### Co-Immunoprecipitation

Co-immunoprecipitation (Co-IP) was performed as a previous study (Adhikary et al., 2016). Briefly, the cells were soaked in lysis buffer, and specific antibodies were adopted to perform immunoprecipitation. For DNase I co-immunoprecipitation, 500 µg of lysate was digested in DNase I for 1 h at 37 °C. The reaction was broken by adding 5  mM EDTA. We used the DNA-free lysate for immunoprecipitation with specific antibodies. After all the reaction ended, we adopted immunoblotting to analyze immunoprecipitants.

### Luciferase Reporter Assays

The Dual-Luciferase^®^ Reporter Assay System (Promega) was used to perform luciferase reporter assays. The cells from different groups were seeded in 24-well plates (2.0 × 10^5^cells per well) and transfected together with a promoter–reporter gene vector and the pRL-TK Renilla luciferase vector. After 48 h of transfection, the cells were harvested and analyzed according to the manufacturer’s instructions. The luciferase activities were normalized to the Renilla luciferase activity of the internal control.

### *In Vivo* Tumorigenicity

Four- to six-week-old female BALB/c mice were provided by the Laboratory Animal Centre of Chongqing Medical University (Chongqing, China) and maintained at Sichuan Provincial People’s Hospital. The protocols were performed after approval by the Animal Ethics Committee according to issued guidelines. 4 × 10^7^ cells mixed with an equal volume of PBS were injected subcutaneously into the region of the right axilla. Tumor sizes were monitored every 3 days using a vernier caliper, and tumor volumes were calculated using the formula [1/2 × long diameter (cm) × short diameter (cm)^2^] and expressed in cm^3^.

### Statistical Analysis

Statistical data acquired from TCGA were merged and processed by R 4.0.0. The Wilcoxon rank-sum test and Wilcoxon signed-rank test were used for comparing the expression levels of KDF1 between OC and the control group. The Kruskal–Wallis test, Wilcoxon rank-sum test, Wilcoxon signed-rank test, and Spearman correlation were used to analyze the relation between KDF1 expression and the grade of clinicopathologic factors. Normal and adjusted Pearson κ^2^ test and the Fisher exact test were used to analyze whether the grade of clinicopathologic factors affects KDF1 expression. Univariate Cox regression analysis and multivariate Cox regression analysis were combined to evaluate the prognostic value of KDF1 expression and other clinicopathologic factors on survival. All assays in our study were performed in triplicate. The data of different groups in each assay were compared by using two-sided Student’s t-test or analysis of variance (ANOVA). A two-sided *p* < 0.05 was considered significant.

## Results

### Identification of Differentially Expressed Genes in OC

**FIGURE 1:**
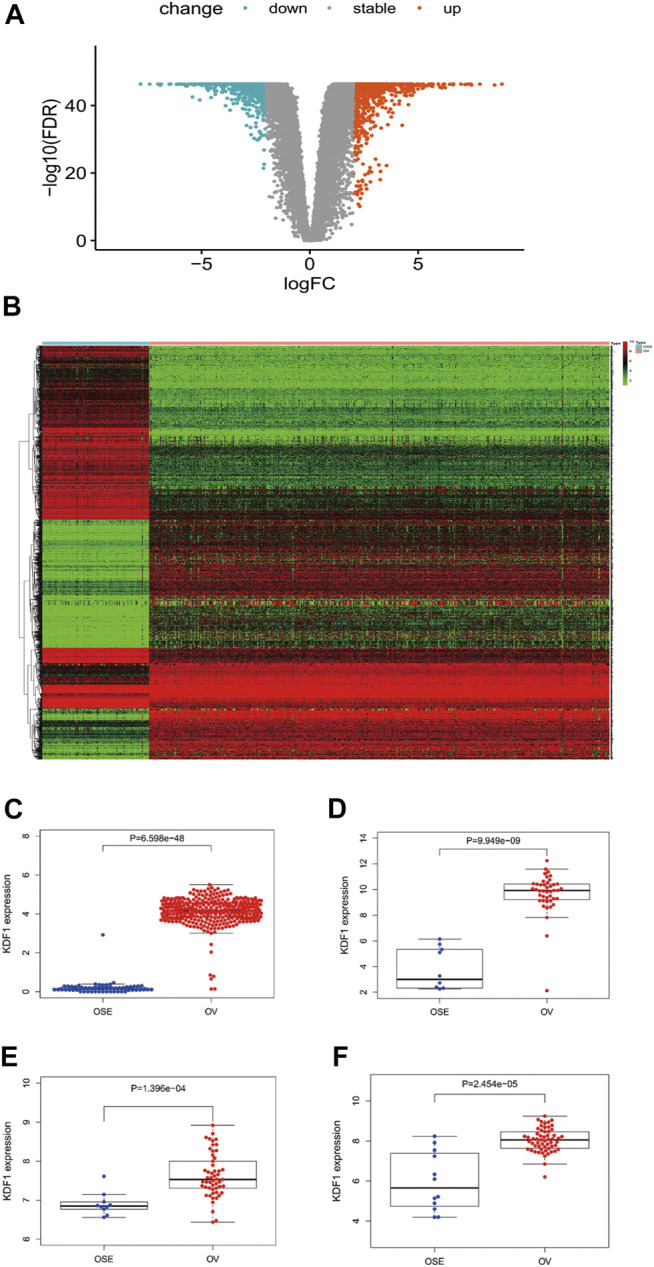
Result of differentially expressed gene analysis. The volcano plot of differentially expressed RNAs in ovarian cancer **(A)**. Heatmap of all differentially expressed genes in ovarian cancer **(B)**. Relationship of expression levels of KDF1 in ovarian cancer tissues and normal ovarian tissues in TCGA **(C)**. Relationship of expression levels of KDF1 in ovarian cancer tissues and normal ovarian tissues was validated by datasets of GSE12470 **(D)**, GSE18520 **(E)**, and GSE66957 **(F)** from the GEO database.

Based on the cutoff criteria (|logFC| <1.5 and adjusted *p* < 0.05), we used the DESeq2 package in R (Love et al., 2014) to analyze the HTSeq-counts data from TCGA. DEG expressions were illustrated by a heatmap (Figure 1A). DEGs included 2008 differentially expressed RNAs (1,017 upregulated and 991 downregulated) (Figure 1B). Differential expression analysis between normal and OC groups indicated KDF1 was expressed significantly higher in OV than normal ovarian tissue (Figure 1C). KDF1 mRNA expression also exhibited significantly increased in GSE12470 (Figure 1D), GSE18520 (Figure 1E), and GSE66957 (Figure 1F).

### Functional Enrichment Analysis of Differentially Expressed Genes and Protein–Protein Interaction Analysis Results

**FIGURE 2:**
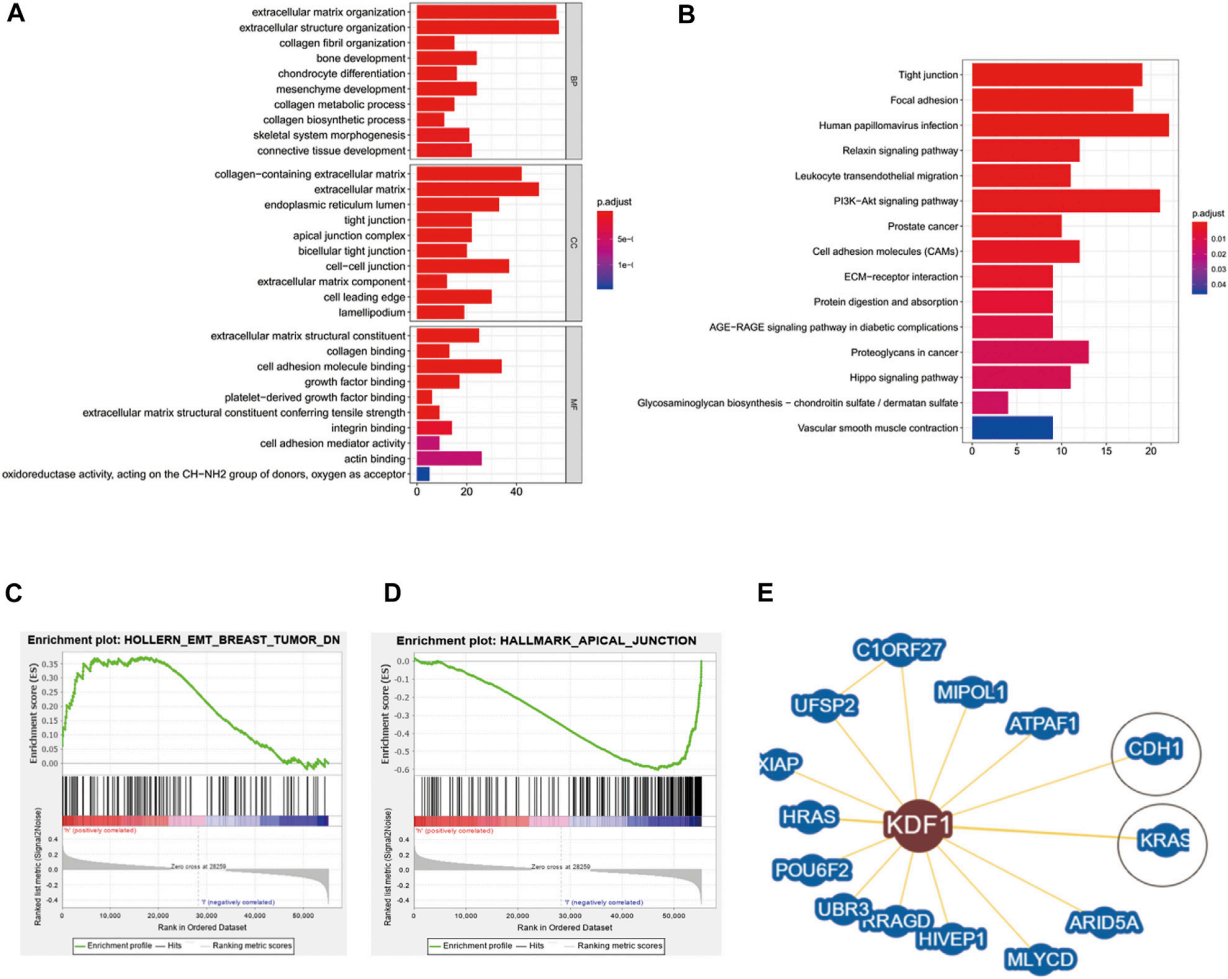
Result of enrichment analysis and protein–protein interaction analysis of KDF1 in ovarian cancer. The result of enrichment analysis from GO **(A)**, KEGG **(B)**, and GSEA **(C)**. The KDF1 interacting protein was predicted by Biogrid **(D)**.

We performed GO and KEGG enrichment analyses of KDF1-associated DEG functions in OC. The GO results displayed that KDF1-associated DEGs had significant regulation on extracellular matrix organization, extracellular structure organization, collagen fibril organization, and bone development in the biological process. Moreover, they also related collagen-containing extracellular matrix, extracellular matrix, endoplasmic reticulum lumen, tight junction, apical junction complex, and cell–cell junction of the cellular component. Extracellular matrix structural constituent, collagen binding, cell adhesion molecule binding, growth factor binding, and platelet-derived growth factor binding of molecular functions were also involved in regulating KDF1 interactive genes (Figures 2A,B). KDF1-related signaling pathways were identified by GSEA (Figures 2C,D). The KDF1 interacting protein predicted by Biogrid included CDH and KRAS (Figure 2E).

### The Prognostic Value of Keratinocyte Differentiation Factor 1

**FIGURE 3:**
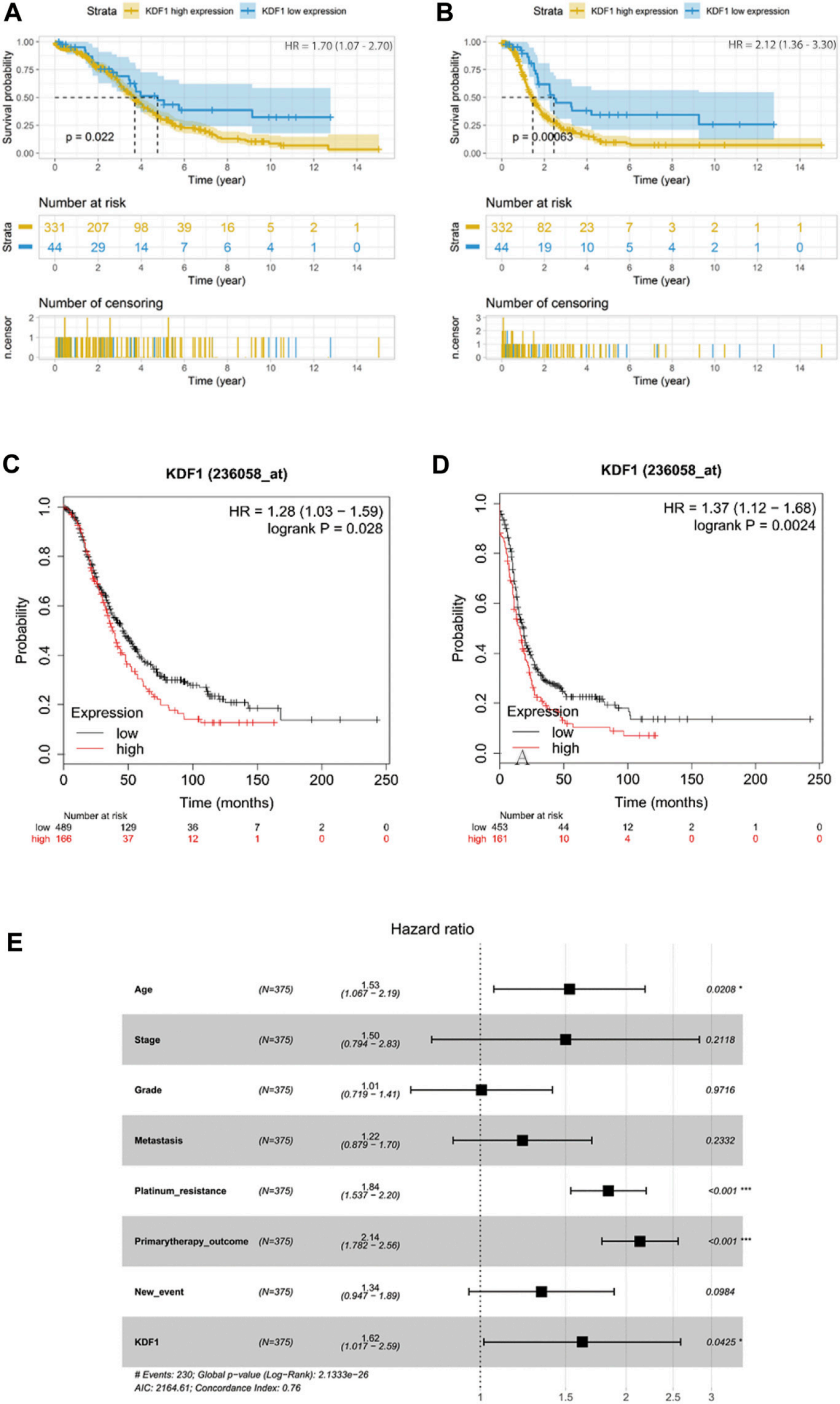
Prognostic value of KDF1 in ovarian cancer. High expression of KDF1 was associated with worse overall survival in TCGA **(A)** and Kaplan–Meier plotter **(C)**. High expression of KDF1 was associated with worse progression-free survival in TCGA **(B)** and Kaplan–Meier plotter **(D)**. Forest plot of the prognostic value of KDF1 in overall survival in TCGA **(E)**.

By analyzing the survival data in the TCGA database, the best cutoff of KDF1 expression was adopted to divide the patients into 331 cases in the high-expression group (1 case missed overall survival data) and 44 cases in the low-expression group. There was a significant difference in the OS–KM curve between high- and low-expression groups (*p* = 0.022) (Figure 3A). The same cutoff value was adopted to divide all patients into 332 cases in the high-expression group and 44 cases in the low-expression group. There was a significant difference in the PFS–KM curve between high- and low-expression groups (*p* < 0.001) (Figure 3B). KDF1 was also input in the online tool Kaplan–Meier plotter to verify the relationship between the expression of KDF1 and the OS or PFS in OC patients. It was found that the difference of the OS–KM curve and the PFS–KM curve of ovarian cancer patients between the KDF1 high- and low-expression groups was statistically significant (*p* = 0.028) (Figure 3C) (*p* = 0.0024) (Figure 3D).

When comparing whether there are differences in different clinical features between high- and low-expression groups of KDF1, it is found that KDF1 is significantly correlated with platinum resistance (*p* = 0.018) and new events, including recurrence and progression of disease (*p* = 0.037) (Table 1).

**TABLE 1:** Difference of clinical characteristics between high- and low-expression groups of KDF1.

Characteristics		KDF1 expression RNAseq		*p* Value
		Low	High	
Metastasis	No	34	282	0.471
	Yes	8	49	
Stage	Stage Ⅰ + Ⅱ	2	21	1
	Stage Ⅲ + Ⅳ	40	310	
Grade	Grade 1 + 2	8	35	0.191
	Grade 3 + 4	34	289	
Platinum_resistance	No	28	182	0.018
	Yes	3	77	
New_event (recurrence or progression of disease)	No	21	109	0.037
	Yes	22	224	
Age	≤50	9	71	0.953
	>50	34	262	
Primarytherapy_outcome	CR	25	186	0.418
	NCR	8	84	

Univariate Cox regression suggested that stage (*p* = 0.05), platinum resistance (*p* < 0.001), and primary treatment outcome (*p* < 0.001) were related to the prognosis of ovarian cancer, and KDF1 had a trend to reach statistical significance (*p* = 0.07) (Table 2). After removing clinical confounding factors by multivariate Cox regression, the results showed that KDF1 (*p* = 0.04), platinum resistance (*p* < 0.001), and initial treatment outcome (*p* < 0.001) were independent risk factors for the prognosis of ovarian cancer. It is suggested that patients with high KDF1 expression have a higher risk of death (HR = 1.62, 95% CI: 1.017–2.59) than those with low KDF1 expression. The forest plot drawn according to Cox multivariate regression after incorporating relevant clinical features suggests that the high expression of KDF1, platinum resistance, and primary therapy outcome are risk factors for low OS in patients with ovarian cancer (Figure 3E).

**TABLE 2:** Relationship between clinicopathologic parameters and expression of KDF1 in 110 cases of ovarian cancer.

	No. of patients	KDF1 expression		*p* Value
—	(*n* = 110)	Low no. (%)	High no. (%)	—
Characteristics	—	—	—	—
Age(years)	—	—	—	>0.05
<50	53	25	28	—
≥50	57	26	31	—
Normal ovarian	30	28	2	<0.05
Cancer tissues	80	23	57	
FIGO stage	—	—	—	—
I/II	45	19	26	<0.05
III/IV	35	4	31	
Grade	—	—	—	—
1	18	14	4	—
2	25	6	19	—
3	37	3	34	—
Grade 2–3 *versus* 1				<0.05

### The Expression of Keratinocyte Differentiation Factor 1 in Ovarian Cancer Tissues and Cell Lines

**FIGURE 4:**
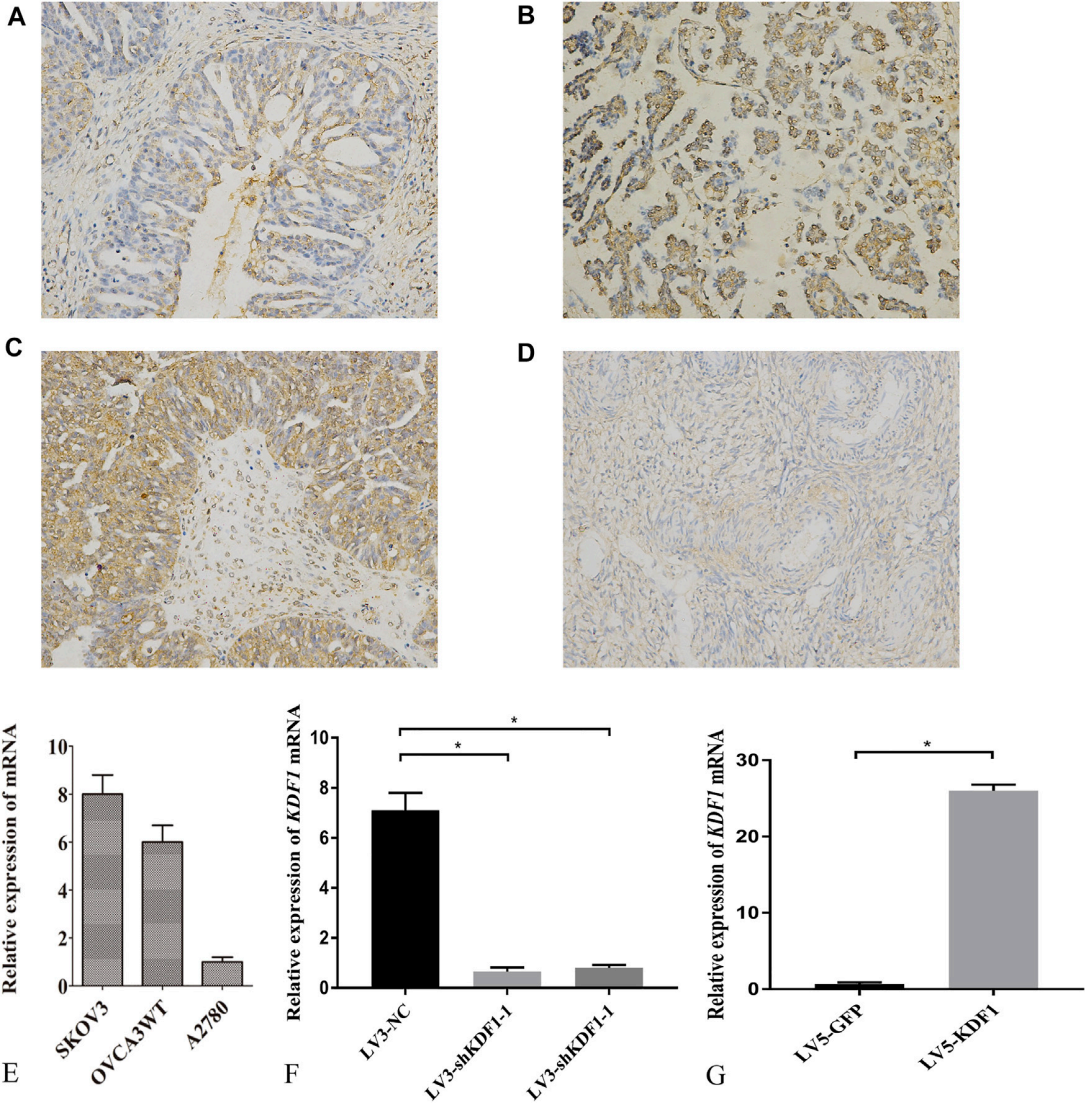
Expression of KDF1 in tissues and cells of ovarian cancer. The expression and location of KDF1 was detected in ovarian cancer tissues **(A–C)** and normal ovarian tissues **(D)**. KDF1 mRNA expression was detected by RT-qPCR **(E–G)**. *indicates *p* < 0.05.

We first detected the location and expression of KDF1 in ovarian cancer tissues using immunochemistry. KDF1 is mainly located in the cytoplasm. The expression of KDF1 was higher in ovarian cancer tissues than that of normal ovarian tissues (Figures 4A–D). The expression of KDF1 in ovarian cancer was relative to grade and stage. The expression of KDF1 was higher in ovarian cancer tissues in advanced stages (stage III/IV) than in those in the early stages (stage I/II; Table 3, *p* < 0.05).

**TABLE 3:** Univariate and multivariate analyses of clinicopathologic parameters in patients with ovarian cancer in TCGA-OV.

Characteristics	HR (95% CI) univariate analysis	*p* Value univariate analysis	HR (95% CI) multivariate analysis	*p* Value multivariate analysis
Age	1.329 (0.935–1.891)	0.11	1.528 (1.067–2.188)	0.02
Stage	2.133 (1.002–4.541)	0.05	1.500 (0.794–2.833)	0.21
Grade	1.222 (0.856–1.744)	0.27	1.006 (0.719–1.409)	0.97
Metastasis	1.263 (0.912–1.751)	0.16	1.222 (0.879–1.699)	0.23
Platinum resistance	2.213 (1.866–2.624)	<0.001	1.838 (1.537–2.200)	<0.001
Primary therapy outcome	2.189 (1.867–2.567)	<0.001	2.136 (1.782–2.560)	<0.001
New event	0.963 (0.702–1.321)	0.81	1.338 (0.947–1.888)	0.10
KDF1	1.537 (0.970–2.435)	0.07	1.623 (1.016–2.591)	0.04

HR: hazard ratio, CI: confidence interval.

**FIGURE 5:**
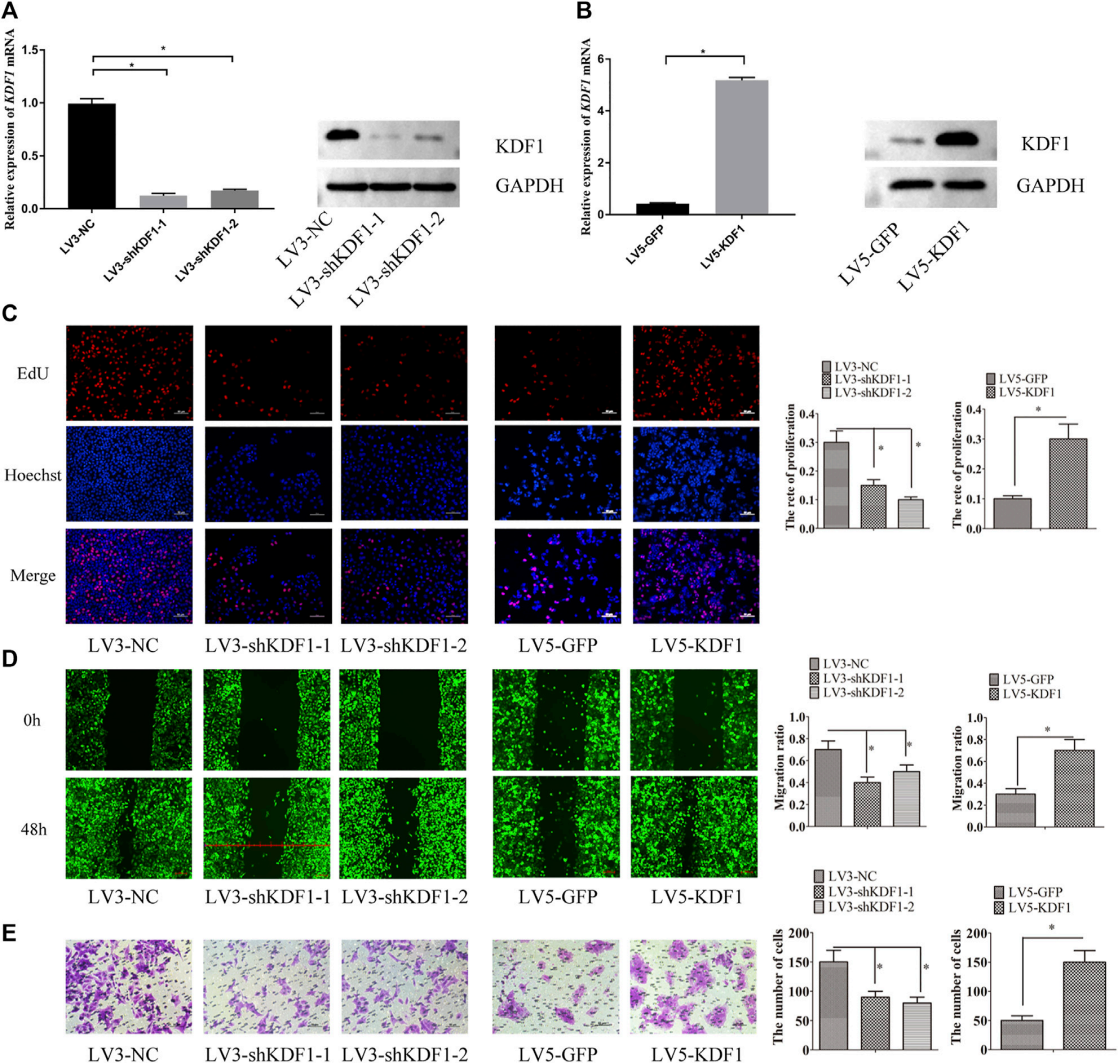
KDF1 promoted proliferation, migration, and invasion of ovarian cancer cells. KDF1 protein expression examined by Western blot **(A,B)**. The cell proliferation ability was detected by EdU assay **(C)**. The cell migration ability was detected by wound scratch assay **(D)**. The cell invasion ability was detected by transwell assay. Error bars represent the standard error **(E)**. *indicates *p* < 0.05.

Furthermore, the staining intensity correlated with the tumor grade (grades 2–3 *versus* 1, Table 3, *p* < 0.05). To investigate the function of KDF1 in ovarian cancer, we screened the mRNA expression of KDF1 in ovarian cancer cell lines. The mRNA expression of SKOV3 and OVCA3WT was higher than that of A2780 (Figure 4E). The gene-silencing and gene-overexpressing efficiency were verified by semiquantitative real-time PCR analysis (Figures 4F,G). Then the KDF1 protein expression in different groups was examined using the Western blot (Figures 5A,B).

### Keratinocyte Differentiation Factor 1 Promoted Proliferation, Migration, and Invasion of Ovarian Cancer Cells

We found that the proliferation, migration, and invasion ability of SKOV3 cells were inhibited after the silencing of KDF1. By contrast, the proliferation, migration, and invasion ability of A2780 cells were elevated after ectopic expression of KDF1 (Figures 5C–E).

### Keratinocyte Differentiation Factor 1 Participated in the Wnt/β-Catenin Pathway in Ovarian Cancer

**FIGURE 6:**
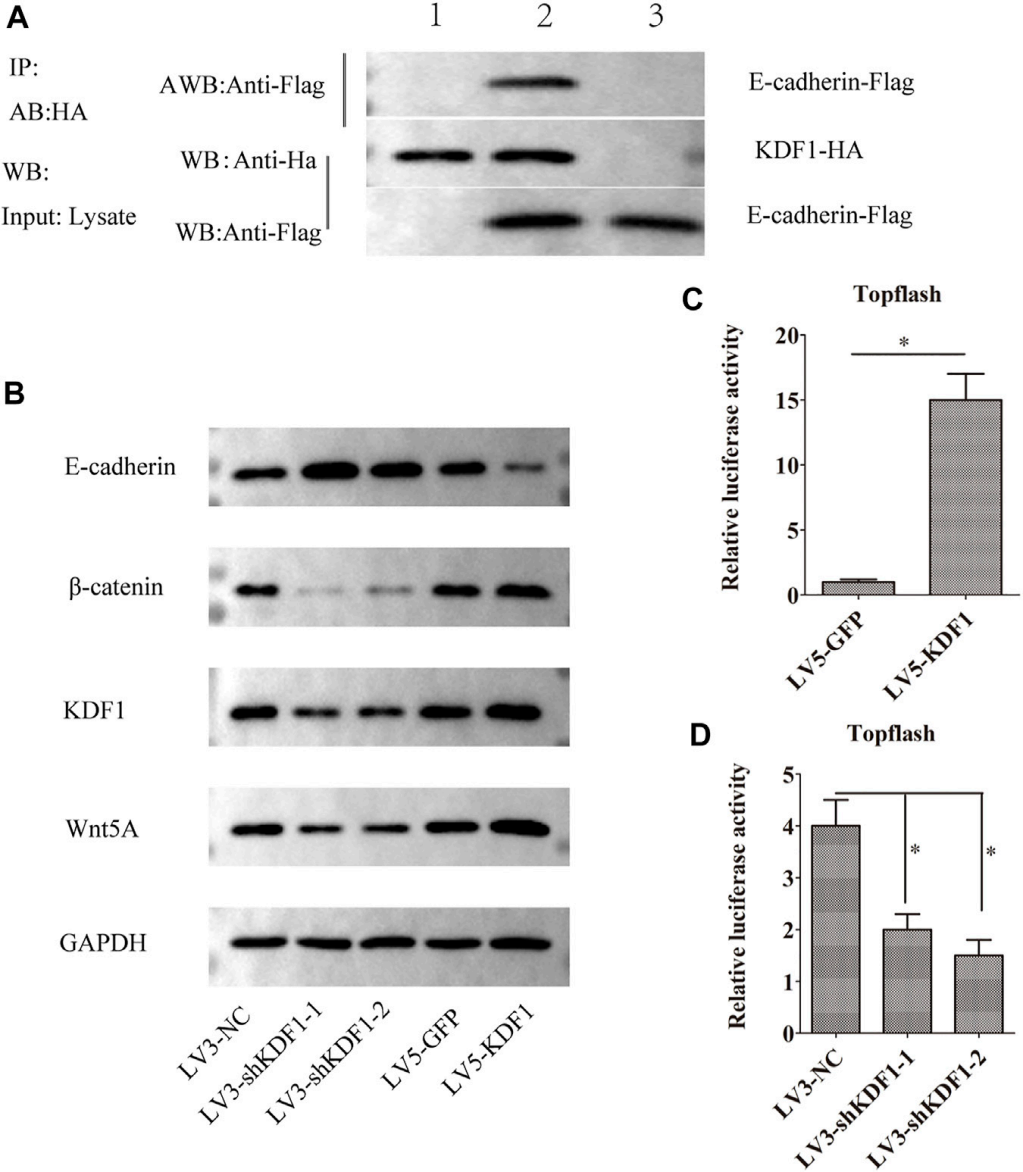
KDF1 participated in the Wnt/β-catenin pathway in ovarian cancer. The Co-IP experiment detected the interaction between KDF1 and E-cadherin protein **(A)**. E-cadherin, Wnt5a, β-catenin, and KDF1 expression was detected using Western blot **(B)**. The regulation of KDF1 on the Wnt pathway was tested using dual luciferase reporter gene experiments **(C,D)**. Error bars represent the standard error. *indicates *p* < 0.05.

As predicted, the interaction between KDF1 and E-cadherin was identified by the Co-IP assay. The cells were co-transfected with Flag-E-cadherin, HA-KDF1, and control group was established simultaneously and harvested 24 h later. Anti-HA antibodies pulled the interaction proteins. Then, they were detected by anti-Flag antibodies. The Western blot displayed that Flag bands could not be detected in the cells transfected with Flag-E-cadherin (lane 1) or HA-KDF1 (lane 3) only. However, it can be detected in cells co-transfected with Flag-E-cadherin and HA-KDF1 (lane 2), indicating that the interaction between KDF1 and E-cadherin may exist *in vivo* (Figure 6A). So, we guessed that KDF1 may regulate the Wnt/β-catenin pathway. Indeed, TOP-flash luciferase activity indicated the level of β-catenin and Wnt5A protein was increased in A2780 cells after ectopic expression of KDF1 (Figures 6B,C). In contrast, silencing KDF1 decreased TOP-flash luciferase activity, the expression of β-catenin, and Wnt5A protein in SKOV3 cells (Figures 6B–D).

### Silencing Keratinocyte Differentiation Factor 1 Inhibited SKOV3 Cells Growth *In Vivo*

**FIGURE 7:**
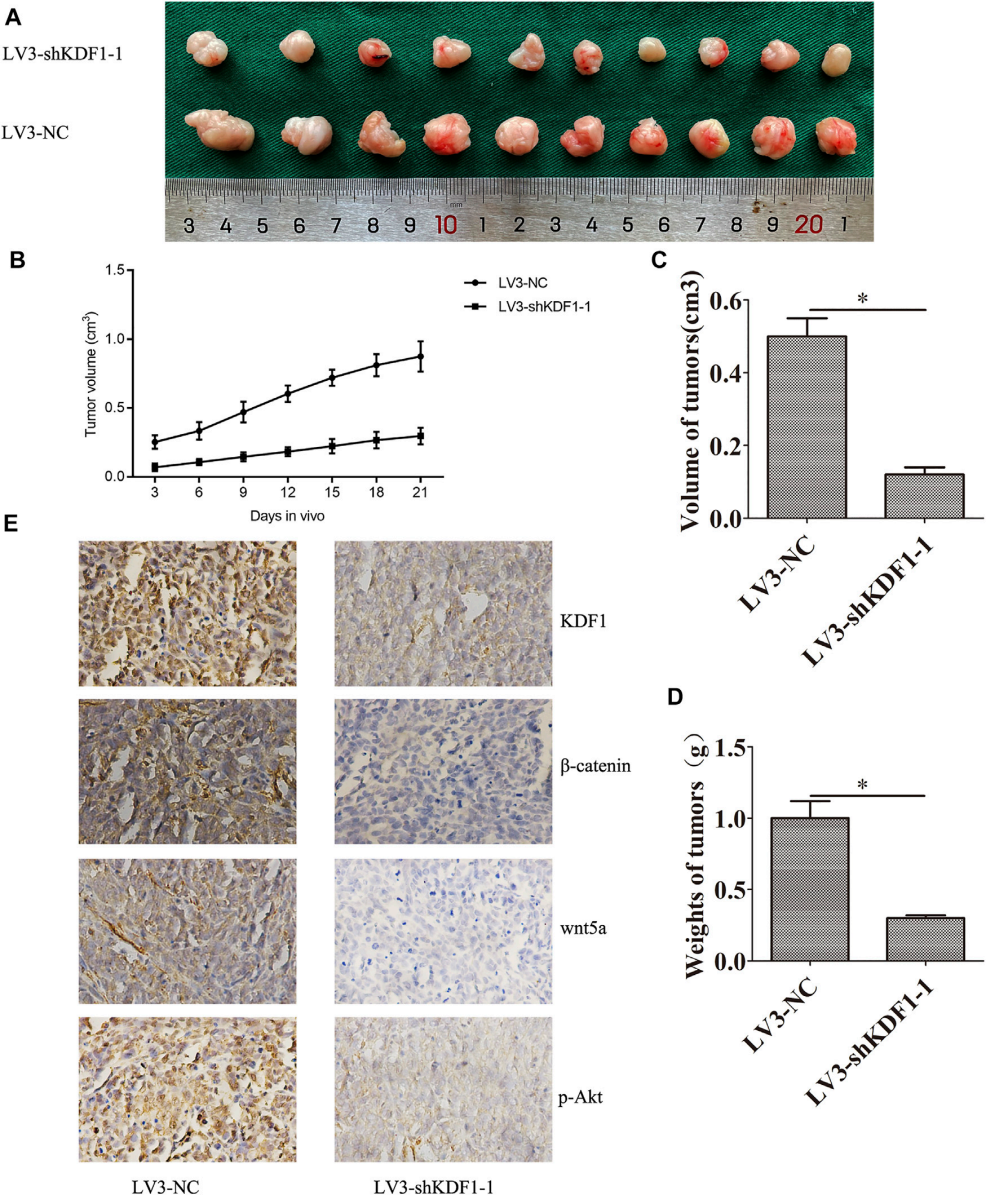
Silencing KDF1 inhibited SKOV3 cells’ growth *in vivo*. The picture of xenograft in two groups **(A)**: the growth curve of tumor in two groups **(B)**, the volume of tumor in different groups **(C)**, the weight of tumor in different groups **(D)**, and the expression of KDF1, Wnt5A, p-AKT, and β-catenin was detected by immunohistochemistry **(E)**. Error bars represent the standard error. *indicates *p* < 0.05.

To investigate the effect of KDF1 on SKOV3 cells’ growth *in vivo*, we constructed a nude mouse subcutaneous tumor model. We observed that silencing KDF1 retarded the growth of SKOV3 cells *in vivo* (Figures 7A–D). The tumor weight and volume were reduced after silencing KDF1. We also observed that the expression of KDF1, Wnt5a, p-AKT, and β-catenin was decreased after silencing KDF1 (Figure 7E).

## Discussion

In this study, bioinformatics analysis of sequencing data from TCGA was performed to gain a deeper understanding of the potential function of KDF1 in OC and to guide further research in OC. Elevated KDF1 expression in OV was associated with advanced clinical pathologic features (new events including recurrence or progression of the disease), poor prognosis, and survival time, which suggested that KDF1 is a potential prognostic and diagnostic marker deserving further research to validate.

Therefore, we used GO and KEGG databases to analyze the gene function of KDF1 and found that KDF1 was associated with the extracellular matrix, tight junction, and other factors. The composition and structure of the extracellular matrix are regulated to control cell behavior and differentiation. If extracellular matrix dynamics is dysregulated, it will lead to cancer and other diseases (Walker et al., 2018). The tight junction is one of the components of the cell junction complex, which includes tight junction, adhesive junction, and desmosomes. It maintains tissue integrity and promotes cell polarity during epithelial cell–cell junction. The tight junction is the critical intercellular junction to establish the epithelial barrier and maintain epithelial polarity (Otani and Furuse, 2020). Extracellular matrix and tight junction are related to tumor invasion and metastasis.

The enrichment of the GSEA gene revealed that high expression of KDF1 was related to the EMT process of breast cancer and was negatively correlated with the cell apical junction process. EMT is a conserved evolutionary cell development program. It participates in cancer by enhancing cell fluidity, invasiveness, and resistance to apoptosis stimulation, and endows cancer cells with metastatic characteristics (Mittal, 2018).

The PPI network analysis of KDF1 indicated that the essential protein E-cadherin might interact with KDF1. Cadherin is a calcium-dependent cell adhesion protein. E-cadherin was a key in establishing and maintaining polarized and differentiated epithelial cells through intercellular adhesion complexes. It participated in regulating epithelial cell adhesion, migration, and proliferation (Meigs et al., 2002).

Elevated KDF1 expression in OV was associated with advanced clinical pathologic features (platinum resistance, new events including recurrence and disease progression), poor prognosis, and survival time. Furthermore, in univariate and multivariate Cox regression analyses, we found that KDF1 was an independent prognostic factor after removing confounding factors, which showed a higher predictive value than many other clinical variables. Our results suggested that KDF1 is a potential prognostic and diagnostic marker deserving further clinical validation.

Bioinformatics research led us to see the strong potential of KDF1 in the occurrence, progression, and prognosis of ovarian cancer, and guided our mechanism research direction into the next stage.

Then, we confirmed that KDF1 is highly expressed in ovarian cancer tissues in the immunohistochemistry assay. High expression of KDF1 was related to tumor stage and histological grade. Our study confirmed that the expression of KDF1 can regulate the phenotype and function of ovarian cancer cells. These effects of KDF1 were further verified by affecting xenograft tumor growth in nude mice. These data indicated that KDF1 was a potential diagnostic marker and therapeutic target for ovarian cancer. Those results suggested that KDF1 is related to cancer progression.

Moreover, we proved that KDF1 could interact with E-cadherin and participate in the Wnt signaling pathway to regulate the EMT process. The activation of the canonical Wnt/β-catenin pathway promoted proliferation and invasion of ovarian cancer (Arend et al., 2013). The Wnt/β-catenin pathway is vital in cell survival and has been implicated in the mechanism of chemoresistance of ovarian cancer (Yamamoto et al., 2019). Research shows that the Wnt/β-catenin pathway is also involved in ovarian tumor angiogenesis (Shoshkes-Carmel et al., 2018) and immune escape (Gregorieff and Clevers, 2005). Wnt activity is related to the grade (Wang et al., 2006), chemoresistance (Chau et al., 2013), and poor prognosis (Jacob et al., 2012; Arend et al., 2013) of patients with ovarian cancer. Meanwhile, the relationship between the Wnt/β-catenin pathway and epithelial-to-mesenchymal transition (EMT) (Arend et al., 2013) has been well documented for a long time.

β-Catenin forms a complex with E-cadherin and has an important role in maintaining epithelial integrity. The deregulation of E-cadherin can accelerate the process of β-catenin entering into the nucleus and activating genes downstream of the pathway (Tafrihi and Nakhaei Sistani, 2017). If the complex is destroyed, it can weaken the adhesion between cells and affect the Wnt signaling pathway (Tian et al., 2011). Wnt5a, which belongs to the Wnt family, is the main regulator of intraperitoneal metastasis and dissemination of ovarian cancer (Asem et al., 2020). In this study, silencing KDF1 can reduce the expression of Wnt5a and β-catenin, while overexpressing KDF1 can increase the expression of Wnt5a and β-catenin. It can be speculated that KDF1 is bound to E-cadherin and played a role in regulating key proteins of the Wnt/β-catenin pathway.

At the same time, dual-luciferase reporter experiment can further determine that the signal pathway reporter is significantly weakened in SKOV3 cells after silencing KDF1, while it is significantly enhanced in A2780 cells after overexpression of KDF1. It was further proved that the expression of KDF1 could affect Wnt/β-catenin pathway activity.

In the immunohistochemistry assay of xenograft tumors in nude mice, compared with the control LV3-NC group, the expression of β-catenin, Wnt5a, and p-Akt in the LV3-KDF1-1 group were decreased. The immunohistochemical results of transplanted tumors proved the results *in vitro* experiments and provided a basis for us to explore the possible interaction between KDF1 and other signal pathways.

We found that KDF1 participated in the Wnt/β-catenin pathway in ovarian cancer by empirical research. Also, we observed that KDF1 directly interacted with E-cadherin. It is suggested that KDF1 and cancer progression, especially through the interaction with E-cadherin, may participate in the Wnt/β-catenin pathway and regulate the EMT process, promoting the proliferation and invasion of OV.

Although our investigation of the relationship between KDF1 and OV helped in further understanding of the vital role of KDF1 in OV, some limitations remained: first, the correlation between KDF1 expression and platinum resistance and other clinical features that needs further investigation. Second, “clinical factors like the details of patients” treatment should be sufficiently considered to clarify the specific role of KDF1 in the development of OV. Third, there are two major shortages in the bioinformatics analysis part of our study. The one is that the treatment information was often inconsistent or even lacking in the public database to clarify the specific role of KDF1 in the development of OV comprehensively; the other is sample size imbalance. We have a smaller number of healthy samples in our control group than that of OV patients in our study; the sample size imbalance may lead to statistical bias. Therefore, future prospective studies are needed to reduce analysis bias.

## Conclusion

In our study, we combined bioinformatics research and *in vivo* and *in vitro* study to systematically prove that KDF1 was a potential oncogene of ovarian cancer. All studies indicated that KDF1 can be a potential diagnostic marker and therapeutic target for ovarian cancer.

## Data Availability

Publicly available datasets were analyzed in this study. This data can be found here: https://portal.gdc.cancer.gov/.
